# Consistent prevalence of asymptomatic infections in malaria endemic populations in Colombia over time

**DOI:** 10.1186/s12936-016-1124-x

**Published:** 2016-02-06

**Authors:** Juan M. Vásquez-Jiménez, Myriam Arévalo-Herrera, Juliana Henao-Giraldo, Karen Molina-Gómez, María Arce-Plata, Andrés F. Vallejo, Sócrates Herrera

**Affiliations:** Clinical Trials Unit, Malaria Vaccine and Drug Development Center, Cali, Colombia; Caucaseco Scientific Research Center, Cali, Colombia; School of Health, Universidad del Valle, Cali, Colombia; Data Management Unit, Caucaseco Scientific Research Center, Cali, Colombia; Molecular Biology Unit, Caucaseco Scientific Research Center, Cali, Colombia

**Keywords:** Malaria, Prevalence, Epidemiology, Colombia, Asymptomatic malaria

## Abstract

**Background:**

Malaria control programmes rely on confirmation of parasite presence in patients’ blood prior to treatment administration. *Plasmodium* parasites are detected mostly by microscopy or rapid diagnostic test (RDT). Although these methods contribute significantly to malaria control/elimination, they are not suitable for detecting the significant proportion of asymptomatic subjects harbouring low levels of parasitaemia, which endure untreated as potential reservoirs for transmission. Malaria prevalence was assessed in endemic regions of Colombia over a 4-year follow-up.

**Methods:**

A series of cross-sectional surveys were conducted between 2011 and 2014 in low to moderate malaria transmission sentinel sites (SS) of Tumaco, Buenaventura and Tierralta municipalities of Colombia. A census was performed and a random sample of houses was selected from each SS prior to each survey. Inhabitants were asked to answer a questionnaire on clinical, epidemiological and demographic aspects, and to provide a blood sample for malaria diagnosis using microscopy and quantitative real time polymerase chain reaction (qPCR).

**Results:**

A total of 3059 blood samples were obtained from all SS, 58.5 % of which were from women and displayed a malaria prevalence ranging from 4 % (95 % CI 3–5 %) to 10 % (95 % CI 8–12 %) in the 4 years’ study period. Almost all malaria cases (n = 220, 97 %) were sub-microscopic and only detectable by qPCR; 90 % of the cases were asymptomatic at the time of blood collection. While Buenaventura and Tierralta had a decreasing tendency during the follow-up, Tumaco had a rise in 2013 and then a decrease in 2014. *Plasmodium vivax* accounted for the majority (66–100 %) of cases in Tierralta and Buenaventura and for 25–50 % of the cases in Tumaco.

**Conclusions:**

This study demonstrates an important prevalence of asymptomatic malaria cases not detectable by microscopy, which therefore remain untreated representing a parasite pool for malaria transmission. This demands the introduction of alternative strategies for diagnosis and treatment, especially for areas of low transmission to reduce it to appropriate levels for malaria pre-elimination efforts to start.

## Background

Malaria is a major public health issue with ~198 million cases and ~584,000 deaths reported worldwide in 2013 [1]. Approximately 427,000 of these cases (~2 %) occur in Latin America, most of which (72.2 %) are reported in South America (Brazil, Venezuela and Colombia) and a minor proportion (10 %) in Central America [2]. During the last ten years Colombia, as well as most of the countries in the region, made significant progress in malaria control, and reported a decrease from ~200,000 cases in 2000 to ~50,000 in 2013 [1]. Despite the ~74 % national malaria reduction experienced between 2000 and 2012, mainly due to the effort of the National Malaria Control Programme (NMCP) with great input from the Global Fund for Aids, Tuberculosis and Malaria (GF), the country still faces a long path towards elimination, as evidenced by the malaria outbreak in 2010 [3]. More than 117,000 cases were reported in 2010 [4] with an apparent association with climate change, illegal agriculture and mining, population migration and inadequacies in NMCP implementation [5].

Microscopic examination of Giemsa-stained thick blood smear (TBS) remains the gold standard diagnostic test used by NMCP before initiation of anti-malarial treatment [6]. Public policies on malaria focus on vector control activities (VCA) and passive case detection (PCD), which limit malaria treatment to symptomatic patients seeking care [7]. However, it is currently accepted that patent symptomatic infections are only the ‘tip of the iceberg’, while asymptomatic subjects represent a large proportion of the population in endemic communities from Asia and Latin America [8, 9]. A significant proportion of these cases harbouring sub-microscopic infections are only detectable by molecular methods, including polymerase chain reaction (PCR), loop mediated isothermal amplification of nucleic acids (LAMP) and high-volume ultrasensitive real-time polymerase chain reaction (HVUSqPCR) [10–15]. In all these studies, molecular techniques provided several-fold higher sensitivity than microscopy and rapid diagnostic tests (RDTs).

Previous cross-sectional studies carried out in Colombia showed that sub-microscopic infections were prevalent across different regions in Colombia (Buenaventura (Bv): 12 %, Tierralta (Ta): 15 %, Tumaco (Tm): 4 %) [16]. Those results highlighted the need to assess the behaviour of these asymptomatic cases with follow-up, particularly considering that neither identification of asymptomatic patients nor their treatment were addressed by NMCP guidelines [6].

Patients with asymptomatic sub-microscopic parasitaemia have previously been shown to transmit infection to *Anopheles* mosquitoes [17, 18] although with lower efficiency than symptomatic patients [17]. However, they may remain asymptomatic during long periods, representing parasite reservoirs for malaria transmission [7]. Unfortunately, little is known about the dynamics of malaria infection in Colombia due of the paucity of active case detection (ACD) activities and the consequent scarcity of data on the nature and magnitude of asymptomatic malaria and its role in transmission. Here, the epidemiology of malaria in a 4-year follow-up is reported, focusing on the description and importance of asymptomatic patients and discussing their potential role in transmission. Despite the overall low to moderate malaria transmission intensity, study sites are among the most endemic of Colombia, where malaria-infected asymptomatic populations are likely to contribute to hamper malaria control efforts and delay malaria elimination.

## Methods

### Study population

This study was performed in ten rural sentinel sites (SS) located in the municipalities of Tierralta (department of Córdoba), Buenaventura (Valle del Cauca) and Tumaco (Nariño). These SS were selected based on the source of malaria cases reported in the Colombian National Surveillance System (SIVIGILA).

Tierralta (Ta) is located in the northwest of Colombia, 51 m above sea level (masl) and ~50 km from the Colombian Atlantic coastline with a mean annual temperature of 27.3 °C. It presents 1144 mm of annual precipitations and has a mean relative humidity (MRH) of 82 % [19]. Its population is an estimated ~97,000 inhabitants, of which ~44 % live in rural areas. Eighty-six per cent of people in Ta are mestizo, 8 % African-American and ~2 % indigenous [20]. Tierralta reported 844 malaria cases to SIVIGILA in 2014 (94.4 % *Plasmodium vivax*, 5.1 % *Plasmodium falciparum* and 0.5 % mixed infection) [21] and is considered a moderate risk zone according to the annual parasite index (API = 8.6 cases/1000 inhabitants). Four rural populations (Los Pollos, El Loro, Tuis Tuis, and La Unión) were surveyed.

Buenaventura (Bv) is a municipality located in west Colombia on the Pacific coast at 7 masl, with a mean annual temperature of 28 °C and a high pluviosity (~ 8000 mm of annual precipitation) [19]. It has a population of ~ 392,000 people (~85 % Afrocolombian, ~10 % mestizos). A total of 334 cases (71.0 % *P. vivax* and 21.0 % *P. falciparum*) were reported by SIVIGILA in 2014 [21], placing Bv as a low risk zone (API = 0.8 cases/1000 inhabitants) [22]. Four SS were selected: Punta Soldado, Zaragoza, Zacarías, and La Delfina.

Tumaco (Tm) is a municipality located in southwest Colombia on the Pacific coast at ~40 km from the border with Ecuador [19]. It has a population of ~195,000 inhabitants [20]. It differs from the aforementioned localities in that *P. falciparum* is by far the most prevalent malaria pathogen. It reported 1309 malaria cases (1.1 % *P. vivax*, 97.6 % *P. falciparum* and 0.3 % mixed infection) in 2014 [21], which places it as a moderate risk zone (API = 6.7 cases/1000 inhabitants). Three SS were selected from this population (Robles, Candelilla and Buchelli).

### Study design

A series of cross-sectional studies were conducted to establish the variation of malaria prevalence in the SS that were considered malaria transmission hotspots, according to data from NMCP between 2011 and 2014. These surveys comprised two stages: (1) a population census was conducted via door-to-door visits with the help of community leaders, in which the amount of houses, inhabitants/house, age and gender were recorded. The sample size (n) was calculated for each SS with a confidence level of 95 % (z = 1.96), error (d) from 2 to 9 % and an estimated prevalence (P) from 2 to 15 % according to the following function $n = \left( {z^{2} } \right)P\left( {1-P} \right)d2$. A set of houses was randomly selected and men and women of any age from the household present at the moment of sampling were asked to answer a questionnaire about symptoms and epidemiologically relevant information. Afterwards, blood samples were obtained (4 mL for adults and 3 mL for <7 years old) by venipuncture and stored in EDTA-containing tubes and two separate microscope slides. The blood was used for microscopic malaria diagnosis by TBS and qPCR. Adults were asked to sign an informed consent (IC) form prior to inclusion, whereas children seven to 18 years old were asked to provide written informed assent (IA). For children <7 years old only the legal tutor was required to sign the IC for inclusion. The study had the approval and oversight of the institutional review board (IRB) of the Malaria Vaccine and Drug Development Centre (MVDC) at all times and it was conducted following the Good Clinical Practice (GCP) guidelines and local regulations.

### Parasite detection by microscopy

Two drops of blood (~100 mL) were deposited on two different microscope slides per volunteer and stained with Giemsa as described [23, 24] to be used as TBS for microscopy analysis. Two experienced malaria microscopists independently examined slides and parasite density (parasites/µL) was estimated after counting parasites per 200 leukocytes. Total parasite load was expressed as the number of parasites/µL and assuming a leukocyte count of 8000/µL. Inter-observer discordances were solved with a reading by a third microscopist. Subjects were considered positive for malaria if two of the three readings were positive.

### Real time quantitative PCR

Molecular detection of malaria infections in whole blood was performed by qPCR targeting the *r18s* gene as previously described [25]. Briefly, DNA was extracted from 200 µL of blood using the QIAmp DNA Blood Mini Kit (QIAGEN, Valencia, CA, USA) and qPCR was carried out with 2 μL of DNA in a total volume of 10 μL, containing 5 μL of TaqMan Universal Master Mix (Applied Biosystems, UK), 0.2 μL of each primer, 0.2 μL Falcprobe, and 0.2 μL Vivprobe. All amplification reactions were performed in a 7500 Real-Time PCR System (Applied Biosystems, USA). A sample was considered negative if there was no increase in the fluorescent signal after a minimum of 40 cycles. Parasitaemia quantification was based on a parasite-specific standard curve made with serial blood dilutions of a reference parasite field isolate. Each reaction plate included a standard curve for parasite quantification.

### Data collection and analysis

Data were captured in the field in paper forms and digitalized and backed-up in REDCap (version 4.1) as described elsewhere [26]. Digital data were stored in duplicates in two online servers. Measures of central tendency and dispersion were calculated for quantitative characteristics, whereas absolute frequencies as well as confidence intervals were used for qualitative characteristics. Inferential statistics, non-parametric and squared Chi test were performed to determine variables of association with sub-microscopic and asymptomatic malaria. Prevalence of malaria by SS and year of study using both microscopy and qPCR was calculated between 2011 and 2014. A threshold for statistical significance was *p* < 0.05 and 95 % confidence intervals were calculated for proportions. R-3.2.2 and MATLAB (version 2011b) were used for statistical analysis. The libraries used in R were dplyr (v0.4.1), ggplot2 (v1.0.1), bitops (v1.0–6), RCurl (v1.95–4.7), Hmisc (v3.16–0), xtable (v1.7–4), maptools (v0.8–36), mapplots (v1.5), plotrix (v3.5–12) and RColorBrewer (v1.1–2).

## Results

### Demographic features

**Fig. 1:**
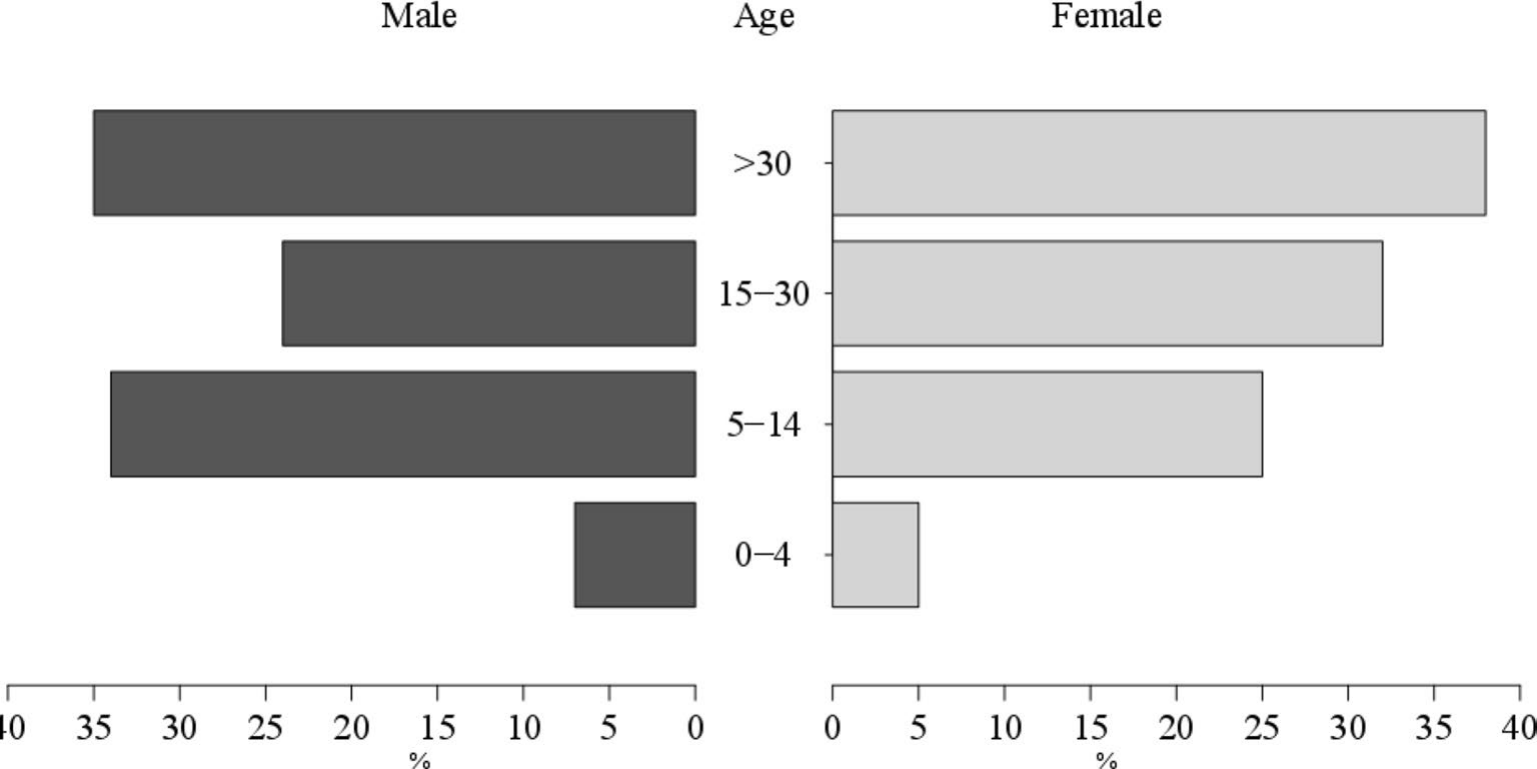
Gender and age group. Percentages of male and female populations are shown for the indicated age groups in *black* and *grey horizontal bars*, respectively

A total of 1169 subjects were evaluated in 2011, 981 in 2013 and 909 in 2014. Census and randomization were performed every year in each site because the studied populations had a high rate of migration. The study did not aim to evaluate the same people at each location every year. Of the total population, 58 % (95 % IC 57–60 %) were women (Fig. 1). Mean ages were 24 (SD = 18) years in Ta, 27 (SD = 19) in Bv, and 29 (SD = 21) in Tm.

### Malaria prevalence

From the total 3059 samples collected from 2011 to 2014, six were positive for *P. vivax* but none for *P. falciparum* when examined by microscopy (Table 1). However, when examined by qPCR, a total of 113 samples from 2011 were found positive (83 *P. vivax*, 25 *P. falciparum*, five mixed), 72 from 2013 (56 *P. vivax* and 16 *P. falciparum*), and 33 from 2014 (22 *P. vivax* and 11 *P. falciparum*), indicating that almost all cases (97 %) were sub-microscopic.

**Table 1:** Prevalence and number of sampled individuals, cases by TBS and qPCR and proportion of species by sentinel site and year

Site	SS	n	2011 TBS + n (%)	qPCR + n (%)	*Pv* n (%)	P*f* n (%)	n	2013 TBS + n (%)	qPCR + n (%)	*Pv* n (%)	P*f* n (%)	n	2014 TBS + n (%)	qPCR + n (%)	*Pv* n (%)	*Pf* n (%)
Tierralta	Tuis tuis	146	0 (0)	15 (10)	14 (93)	1 (7)	40	0 (0)	3 (8)	3 (100)	0 (100)	97	0 (0)	4 (4)	4 (100)	0 (0)
	La Union	126	1 (1)	25 (20)	24 (96)	1 (4)	40	0 (0)	10 (25)	10 (100)	0 (0)	97	0 (0)	6 (6)	2 (33)	4 (67)
	El Loro	–	–	–	–	–	50	0 (0)	3 (6)	3 (100)	0 (0)	97	0 (0)	4 (4)	3 (75)	1 (25)
	Los Pollos	–	–	–	–	–	42	0 (0)	2 (5)	2 (100)	0 (0)	65	0 (0)	3 (5)	3 (100)	0 (0)
	Total	272	1 (0.4)	40 (15)	38 (95)	2 (5)	172	0 (0)	18 (10)	18 (100)	0 (0)	356	0 (0)	17 (5)	12 (71)	5 (29)
Buenaventura	Pta. Soldado	145	0 (0)	23 (16)	13 (57)	9 (39) +	82	0 (0)	0 (0)	0 (0)	0 (0)	48	0 (0)	0 (0)	0 (0)	0 (0)
	Zacarías	163	0 (0)	14 (9)	11 (79)	0 (0) +	160	0 (0)	0 (0)	0 (0)	0 (0)	100	0 (0)	1 (1)	1 (100)	0 (0)
	La Delfina	129	0 (0)	16 (12)	11 (69)	5 (31)	84	0 (0)	5 (6)	5 (100)	0 (0)	72	0 (0)	1 (1)	0 (0)	1 (100)
	Zaragoza	–	–	–	–	–	61	0 (0)	21 (34)*	21 (100)	0 (0)	89	2 (2)	10 (11)*	8 (80)	2 (20)
	Total	437	0 (0)	53 (12)	35 (66)	14 (26)	387	0 (0)	26 (7)	26 (100)	0 (0)	309	2 (1)	12 (4)	9 (75)	3 (25)
Tumaco	Robles	150	1 (1)	4 (3)	1 (25)	3 (75)	135	0 (0)	15 (11)	6 (40)	9 (60)	88	0 (0)	2 (2)	1 (50)	1 (50)
	Candelilla	160	2 (1)	12 (8)	8 (67)	4 (33)	147	0 (0)	5 (3)	4 (80)	1 (20)	74	0 (0)	0 (0)	0 (0)	0 (0)
	Buchelli	150	0 (0)	4 (3)	1 (25)	2 (50) ++	140	0 (0)	8 (6)	2 (25)	6 (75)	82	0 (0)	2 (2)	0 (0)	2 (100)
	Total	460	3 (0.6)	20 (4)	10 (50)	9 (45)	422	0 (0)	28 (7)	12 (43)	16 (57)	244	0 (0)	4 (2)	1 (25)	3 (75)
Total by year		1169	4 (0.3)	113 (9.7)	83 (73.5)	25 (22.1)	981	0 (0)	72 (7.3)	56 (77.8)	16 (22.2)	909	2 (0.2)	33 (3.6)	22 (66.7)	11 (33.3)

*TBS* thick blood smear microscopy, *qPCR* quantitative polymerase chain reaction, *Pv* *P. vivax*, *Pf* *P. falciparum*

* Statistically significant difference between SS (*p* < 0.05)

+ Buenaventura had four cases of mixed malaria in 2011

++ Tumaco had 1 case of mixed malaria in 201 l

**Fig. 2:**
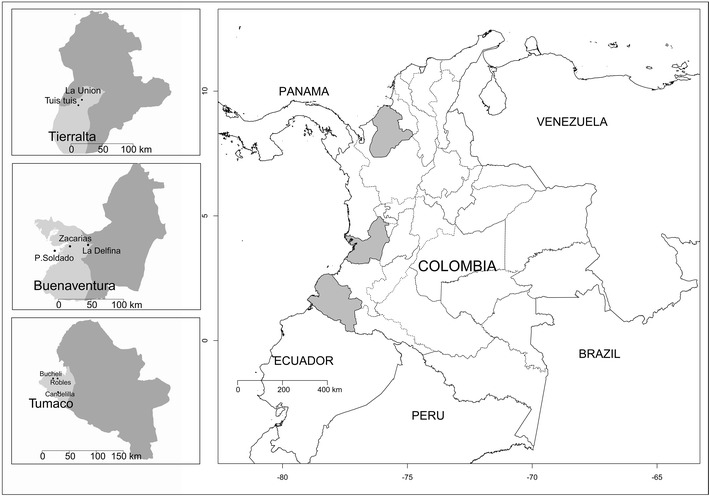
Study sites. Map of Colombia showing the location of the sentinel study sites

Total malaria prevalence as determined by qPCR was 10 % (95 % CI 8–12 %) in 2011, 7 % (95 % CI 5–9 %) in 2013, and 4 % (95 % CI 3–5 %) in 2014. A significant difference between 2011 and 2014 prevalences was found (*p* < 0.001). In contrast, < 0.2 % of the samples were positive by TBS during the whole study. Despite the overall malaria decreasing trend, the proportion of *P. vivax* did not change significantly between 2011 and 2013 (83/113 (73 %, 95 % CI 64–81 %) and 56/72 (78 %, 95 % CI 66–86 %), respectively); but decreased to 22/33 (67 %, 95 % CI 48–81 %) in 2014. The proportion of *Plasmodium* species varied depending on the municipality, with *P. vivax* accounting for the majority (66–100 %) of cases in Ta and Bv and for 25–50 % of the cases in Tm. The study sites are showed in Fig. 2.

Most cases in 2011 were detected in people > 30 years (n = 113, 35 % CI 25–43 %), in 2013 the majority of cases were in young adults (15–30 years/old) (n = 72, 36 % CI 25–48 %) and in 2014 the main age group was children five to 14 years old (n = 33, 42 % CI 25–60 %). There was a decreasing trend with age along the study period, however no significant differences were found. Tierralta showed the highest percentage of positive cases in children five to 14 years old, including all years (n = 75, 34 %) with significant differences with the other age groups (0–4 = 5 %, 15–30 = 32 %, >30 = 28 %) (*p* < 0.001). In Bv and Tm people >30 years showed the highest percentage of cases, 33 % for Bv (n = 91, 0–4 = 13 %, 5–14 = 27 %, 15–30 = 26 % *p* < 0.001) and 44 % in Tm (n = 52, 0–4 = 6 %, 5–14 = 21 %, 15–30 = 29 % *p* < 0.001) with significant differences.

### Parasitaemia

No significant differences were found for median parasitaemias among the study sites or throughout the follow-up (Table 2). However, parasitaemia was significantly higher in subjects with *P. falciparum* infections than in those with *P. vivax* (*p* < 0.001). Median parasitaemia values found per site respectively for *P. vivax* and *P. falciparum,* respectively, were: four and nine parasites/µl in Ta, five parasites/μL for both species in Bv, and 41 and 142 parasites/μL in Tm. There was a low negative correlation between age and parasitaemia for *P. vivax* in Bv, although not significant. Additionally, the correlation between patient-reported history of number of previous malaria episodes and current parasitaemia in *P. falciparum* was also low and negative. People who reported having suffered more malaria episodes had lower parasitaemia than those who reported fewer episodes. No significant differences were found between time to previous episode and parasitaemia.

**Table 2:** Summary statistics for parasitaemia values

Area	Year	Mean P*v*/P*f*	SD P*v*/P*f*	Median P*v*/P*f*	Min P*v*/P*f*	Max P*v*/P*f*
Ta	2011	55.2/87	162.8/119	1.8/87	0.1/3	813/171
	2013	34.4/–	42.1/–	16.5/–	1/–	152/–
	2014	7.8/13	10.4/16	2/9	1/1	33/40
Bv	2011	54.2/61	180.1/220	2/6	0.1/0.2	833.4/940
	2013	48.3/–	60.5/−	26.5/−	1/–	254/–
	2014	213.3/4	622.8/2	2/5	1/1	1874/5
Tm	2011	60.4/221	39.7/296	51/120	8/27	116/1021
	2013	39.2/484	35.6/1204	28.5/154	1/5	96/4958
	2014	1/37	–/33	1/33	1/6	1/71

Parasites per µl by species for each area and year. *Pv* *P. vivax*, *Pf* *P. falciparum*

### Asymptomatic cases

A total of 195 of the 220 positive patients (95 % CI 84–92 %) did not report malaria symptoms: 143 for *P. vivax*, 47 for *P. falciparum* and five harboured mixed infections. Of the 214 cases exclusively positive by qPCR, 192 were asymptomatic (90 %, 95 % CI 85–93 %). Although the asymptomatic malaria prevalence trend decreased steadily in Bv with time, in Ta and Tm it presented variations, with a decline in 2013 and a slight increase in 2014. The most common symptoms in people infected with malaria were fever, headache and chills. Eighteen individuals had parasitaemias <100 parasites/mL and presented the same symptoms, which were probably due to other etiologies, although they were not further investigated in this study. A total of 261 volunteers were symptomatic without detectable infection by qPCR. These latter volunteers were possibly infected with other agents presenting overlapping malaria symptoms.

## Discussion

This study shows a malaria decreasing trend in the study areas from 9.7 % in 2011 to 3.6 % in 2014, as determined by qPCR, despite some variability per year. Although the SS are not necessarily representative of all departments of the country, these findings reflect the overall national malaria trend for the same years, as established on NMCP records based on PCD and the use of microscopy and RDT [21]. As expected, the ACD surveys showed that a significantly lower number of cases were detected by microscopy compared to qPCR. The parasite species proportions remained similar with a significantly higher overall prevalence of *P. vivax* (between 67 and 78 %) in Ta and Bv; and higher *P. falciparum* prevalence in Tm. The different parasite species distribution is most likely due to the population structure in those regions. The population in Tm is likely to have a higher proportion of Duffy negative (Fy-) individuals, probably protecting them against *P. vivax* infections [27].

The situation in Zaragoza (Bv) where the community is highly involved in illegal mining activities is alarming as compared to the other communities. In 2013, 34 % of people examined by qPCR in this SS, corresponding to 81 % of the cases found in Bv by ACD, were infected with *Plasmodium,* most of whom (76 %) were asymptomatic. However, NMCP reports in Bv indicated that only ~40 % of the malaria cases detected by PCD originated in Zaragoza [21]. Comparison of the prevalence in Zaragoza with other SS in Bv shows a statistically significant difference (*p* < 0.001). This could be explained by persistence of a high proportion of asymptomatic individuals in the community and possibly the maintenance of the vector reservoir created by mining activities. Almost 99 % of the asymptomatic cases displayed sub-microscopic parasitaemia only detectable by qPCR in the three study sites, which confirms a previous report using PCR in 2006 and 2007 from Ta and Tm [28, 29]; these cases remain overlooked by the NMCP which only reports TBS detectable cases in PCD. In this study, qPCR detected 36-fold more cases than microscopy.

Interestingly in other localities of Bv, such as Zacarias and Punta Soldado, as well as Candelilla in Tm, malaria has notably diminished to <1 % as detected by qPCR in 2014. These findings indicate an important step towards malaria elimination in these sites. It is noteworthy that while the *P. vivax* proportion decreased compared to that of *P. falciparum,* the prevalence of the latter remained low but stable. This finding is in agreement with NMCP data and also evidenced by Tm data, reporting 1019 malaria cases in 2011 by ACD, 50.5 % caused by *P. vivax,* while in 2014 only 1.1 % of 1309 total cases were caused by this species [21]. This suggests that malaria control activities during this period did not affect *P. falciparum* transmission but had an important impact on *P. vivax*.

It is also interesting that the prevalence of asymptomatic patients by ACD decreased in parallel with acutely ill patients demanding diagnosis and treatment (PCD). Even though the same volunteers were not examined at every time point, a malaria decreasing trend along with asymptomatics that are not treated led to the presence of sub-microscopic parasite pools for long periods, creating a source of infection for mosquitoes and therefore of malaria transmission. Because of the study design, the period for which these infected volunteers remained asymptomatic could not be determined. However, even if these subjects were to develop symptoms, it is highly likely that they would remain untreated, as there is no ACD in these areas and only a minor fraction of the study population that was symptomatic and qPCR positive sought care. In view of malaria elimination, it is of great concern that TBS and RDT show a sensitivity and specificity limited to 50–67 % [11, 30, 31]. The demonstrated persistence and consistency of the prevalence of asymptomatic cases over time calls for more sensitive detection methods. Molecular methods, including qPCR, with sensitivity and specificity of 99–100 % are not yet affordable for mass use in the field. Emerging malaria elimination programmes need to address these constraints.

The use of molecular methods and radical cure of asymptomatics would significantly accelerate elimination, despite the fact that some individuals may remain infected with parasitaemias not detectable even by qPCR. An ongoing study at the MVDC on the infectivity of asymptomatic subjects has indicated that asymptomatic and qPCR-negative subjects living in endemic villages are able to infect *Anopheles albimanus* mosquitoes. In addition it has been shown that qPCR techniques using higher sample volumes can detect cases that are missed by qPCR performed on standard filter paper stored samples or lower blood volumes [32]. Even though official malaria prevalence reports indicate a decreasing trend due to increasing control efforts [33], in low transmission areas molecular testing with higher sensitivity and specificity is needed, as evidenced in this study by 90 % of the cases that were qPCR positive were asymptomatic. This study also underscores the importance of conducting active surveillance not only to more accurately determine malaria prevalence in endemic regions, but also for curative treatment aiming at malaria reservoir elimination. A good molecular technique candidate for use in field at the moment as part of an active case detection strategy is LAMP. This technique has been studied previously in the country for malaria diagnosis and was shown to detect very low parasite densities. LAMP is more cost-effective than qPCR, however, as qPCR it requires trained staff and laboratories. Although introduction of molecular techniques as routine in diagnosis as part of active search remains a challenge, this study supports this need based on the reported persistence of asymptomatic cases over time which can be detected only by molecular approaches.

## Conclusions

There is prevalence of malaria in endemic regions in Colombia considered to be of low and moderate transmission. The fact that almost all (97 %) of the cases were not detectable by TBS, which is the most widely NMCP-used diagnostic method underscores the need to consider introducing molecular methods for malaria diagnosis. In addition, curative treatment should follow a positive diagnosis, even if asymptomatic, in order to effectively decrease malaria prevalence and further transmission, particularly in view of current plans to accelerate elimination activities [34].
